# Genome Sequence of Tumor Virus X, a Member of the Genus *Protoparvovirus* in the Family *Parvoviridae*

**DOI:** 10.1128/genomeA.00758-14

**Published:** 2014-07-31

**Authors:** Ellen M. Vollmers, Anthony D’Abramo, Susan F. Cotmore, Peter Tattersall

**Affiliations:** a Medical Scientist Training Program, Yale University Medical School, New Haven, Connecticut, USA; b Department of Laboratory Medicine, Yale University Medical School, New Haven, Connecticut, USA; c Department of Genetics, Yale University Medical School, New Haven, Connecticut, USA

## Abstract

The orphan parvovirus tumor virus X (TVX) has potent oncolytic activity. Compared to other viruses from the species *Rodent protoparvovirus 1*, TVX has a 111 nucleotide deletion in its nonstructural (NS) gene, a 24 nucleotide insertion in VP1, and a 93 nucleotide repeat initiating from the C-terminus of the capsid gene.

## GENOME ANNOUNCEMENT

Rodent parvoviruses from the genus *Protoparvovirus* are inherently oncosuppressive and oncolytic in human tumors, while failing to kill untransformed human cells (1). This inherent oncotropism coupled with the ability of parvovirus-induced cell death to stimulate an anti-tumor immune response make the viruses a potentially powerful weapon for fighting cancer (2–4).

Tumor virus X (TVX) was isolated in 1965 from a permanent human amnion cell line and shown to have limited serological overlap with rodent parvoviruses Kilham rat virus (KRV) and H1 (5). Stored frozen and unstudied for more than 40 years, an isolate obtained from the University of Bern, Switzerland, was recovered in 2009 by amplification in HeLa cells and included with 6 other rodent parvoviruses in a screen to identify the best candidates for oncolysis of human malignant melanoma. TVX demonstrated an unsurpassed ability to kill a restricted subset of melanoma cell lines starting from a very low MOI (6).

Primers used to amplify the first TVX sequence from the viral stock, HelFOR (5′ CCAGACAGTTACATTGAAATGATGG 3′) and HelREV (5′ GTGTTGGTTCAATCTGTTTGCTGCC 3′), were selected as regions of exceptional conservation identified by aligning the helicase domains of NS1 proteins encoded by rodent parvoviruses minute virus of mice (MVM) (GenBank J02275), H1 (GenBank X01457), LuIII (GenBank M81888), and KRV (GenBank AF321230). Sequences downstream of this site and extending into the stem of the viral left-end hairpin telomere were then sequenced using the the MVM-based primer MVHLP (5′ TTCACATGGTTGGTCAGTTCTAAAAATGATA 3′). Sequences upstream of the helicase and extending into the right-end viral telomere were determined using forward primers based on acquired TVX sequence and reverse primers selected from regions of high conservation identified by aligning the genomes of 17 viruses and virus strains from the species *Rodent Protoparvovirus 1*, including all of the taxonomically recognized isolates (7). The reverse primers, from most proximal to most distal, were 2kSeqREV 5′ AGAGAAGTACAGGTAAGGAT-3′, 4kSeqRev 5′ AGTCTAGGTTTGTGTTCCA 3′, and PastPshA 5′ CCACCCACCCACCCTAAAAACATAG 3′. In total 4,713 nucleotides of the TVX sequence were obtained, which included the entire viral coding region flanked by PmeI and PshAI restriction sites. This sequence was cloned between MVM terminal hairpins, resequenced in its entirety, and its infectivity was confirmed following transfection into transformed human cell lines.

The TVX genome contains three conspicuous sequence anomalies when compared to other rodent parvoviruses: a deletion, an insertion, and a repeat. TVX has an in-frame, 111 nucleotide deletion within the coding sequence of NS1 and the second exon of NS2, deleting amino acids 587 to 624 of NS1, and 96 to 132 of NS2. This deletion bears striking resemblance to that of an H-1 variant named H-1 dr (8, 9) but is one codon shorter at its 3′ end. TVX also harbors a 24 nucleotide insertion, encoding 8 in-frame amino acids, in the VP1-specific region of the coat protein, and a perfect 93 nucleotide repeat that spans the C-terminus of the capsid gene. The similarity in size between the nonstructural (NS) deletion (111 nt) and the combined VP1 insertion and downstream repeat (117 nt), suggests that the repeat may serve to adjust the genome length to around 5 kb, to ensure efficient packaging and uncoating.

### Nucleotide sequence accession number.

The GenBank accession number of TVX is KJ631100.
